# Expectation effects on brain dopamine responses to methylphenidate in cocaine use disorder

**DOI:** 10.1038/s41398-019-0421-x

**Published:** 2019-02-15

**Authors:** Gene-Jack Wang, Corinde E. Wiers, Elena Shumay, Dardo Tomasi, Kai Yuan, Christopher T. Wong, Jean Logan, Joanna S. Fowler, Nora D. Volkow

**Affiliations:** 1 Laboratory of Neuroimaging, National Institute on Alcohol Abuse and Alcoholism, Bethesda, MD 20892-1013 USA; 2 School of Life Science and Technology, Xidian University, 710071 Xi’an, Shaanxi China; 3 Department of Radiology, New York University, New York, NY 11793 USA; 4 Brookhaven National Laboratory, Upton, NY 11973 USA; 5 National Institute on Drug Abuse, National Institutes of Health, Bethesda, MD 20892 USA

## Abstract

The response to drugs of abuse is affected by expectation, which is modulated in part by dopamine (DA), which encodes for a reward prediction error. Here we assessed the effect of expectation on methylphenidate (MP)-induced striatal DA changes in 23 participants with an active cocaine use disorder (CUD) and 23 healthy controls (HC) using [^11^C]raclopride and PET both after placebo (PL) and after MP (0.5 mg/kg, i.v.). Brain dopamine D2 and D3 receptor availability (D2R: non-displaceable binding potential (BP_ND_)) was measured under four conditions in randomized order: (1) expecting PL/receiving PL, (2) expecting PL/receiving MP, (3) expecting MP/receiving PL, and (4) expecting MP/receiving MP. Expecting MP increased pulse rate compared to expecting PL. Receiving MP decreased D2R in striatum compared to PL, indicating MP-induced striatal DA release, and this effect was significantly blunted in CUD versus HC consistent with prior findings of decreased striatal dopamine responses both in active and detoxified CUD. There was a group × challenge × expectation effect in caudate and midbrain, with expectation of MP increasing MP-induced DA release in HC but not in CUD, and expectation of PL showing a trend to increase MP-induced DA release in CUD but not in HC. These results are consistent with the role of DA in reward prediction error in the human brain: decreasing DA signaling when rewards are less than expected (blunted DA increases to MP in CUD) and increasing them when greater than expected (for PL in CUD reflecting conditioned responses to injection). Our findings also document disruption of the expectation of drug effects in dopamine signaling in participants with CUD compared to non-addicted individuals.

## Introduction

There is growing evidence that the context under which a drug is administered can affect the behavioral, physiological, and the neurochemical response to the drug. For example, the response to drugs of abuse is affected by expectation, which in turn is sensitive to prior drug experiences^1–3^. This is modulated in part by dopamine (DA), which is a neurotransmitter involved with reward and expectation of reward. Animal studies have shown that expectation affects the responses to drugs of abuse^1–3^. For example, cocaine-induced increases in DA and its behavioral effects are greater when animals receive the drug in an environment where they expect it than when they do not^2^ or when they self-administer it than when it is administered by the investigator^3^. Similarly, in human cocaine abusers the behavioral and regional brain metabolic responses to stimulants have been shown to be stronger when stimulants are expected versus when placebo (PL) is expected^4^. The dopaminergic system has also been shown to be involved in the PL-induced expectation of therapeutic benefit including analgesia^5^. Similarly, the involvement of the dopaminergic response has been documented for PL responses in Parkinson’s disease: both to medications^6^ and to repetitive transcranial magnetic stimulation^7^. That is, patients who responded to PL (reporting clinical improvement) had greater DA release in the striatum than those who did not^6,7^.

We have studied the effects of expectation on the effects of the stimulant drug methylphenidate (MP) on regional brain glucose metabolism and on behavior^4,8^. MP, like cocaine, increases DA by blocking DA transporters (DATs); hence, its effects are a function of the level of DAT blockade and the rate of DA release (reflects DA cell activity)^9^. In healthy volunteers, we showed that the expectation of receiving MP while receiving PL significantly activated brain glucose metabolism in the nucleus accumbens (NAc) and ventral cingulate cortex (Brodmann area 25), and effects were strongest in subjects who, because of experimental randomization, had not experienced MP before^8^. These results corroborated the involvement of the NAc and the ventral cingulate in processing expectation for “uncertain drug effects.” However, the extent to which these regional changes reflect the involvement of DA in processing expectation of drug effects rather than non dopaminergic signaling (i.e glutamatergic signaling^10^) needs further investigation. Understanding the neuronal signals underlying expectation responses in addiction and specifically that of DA could help guide development of targeted pharmacotherapeutic interventions.

Further, the characterization of how expectation influences drug responses in non-addicted and addicted individuals might also help guide prevention interventions. Indeed, while expectations might differently impact the effects of drugs of abuse in controls^8^ and in addicted individuals due to conditioning^4,11^, there is also evidence that they might be influenced by genetics^12^. For example, a recent study showed that individuals with family history of alcoholism showed stronger striatal DA release when expecting to receive alcohol, suggesting that genetics influence the sensitivity to expectation; although surprisingly these responses did not differ between controls and alcohol use disorder (AUD) participants^13^. Inasmuch as drug-induced DA increases trigger conditioning to the drug and its cues, one would have predicted that in addicted individuals the expectation effects on DA signaling would be stronger than in controls^14^. However, once conditioning occurs, DA neurons stop firing upon receipt of the reward and instead fire when exposed to the conditioned cues (that predict the reward) or to an unexpected larger reward, whereas they inhibit their firing when the experience of the reward does not materialize or is less than expected^15^. Therefore, it is likely that the differences in DA responses between addicted and non-addicted individuals will depend on whether the expectation is for PL versus stimulant drugs.

To test this possibility, we studied 23 subjects with active cocaine use disorder (CUD) and 23 healthy non-drug- dependent control subjects (HC) with Positron Emission Tomography (PET) and [^11^C]raclopride, a DA D2/D3 receptor (D2R) ligand that is sensitive to competition with endogenous DA^16^. Each subject underwent 4 [^11^C]raclopride scans: (1) PL/PL: expecting PL and receiving PL; (2) PL/MP: expecting PL and receiving MP; (3) MP/PL: expecting MP and receiving PL; and (4) MP/MP: expecting MP and receiving MP. We hypothesized that expectation of receiving the drug would increase DA release in striatum (i.e., lower D2R availability) when compared with unexpected and that because of conditioning in CUD we hypothesized that the difference from expectation (expected MP versus unexpected MP) would be greater in CUD than in HC in line with our previous findings that expectation of MP increased brain glucose metabolism in CUD versus HC^4^, even though the overall dopaminergic effects of MP would be greater in HC than in CUD regardless of condition^17^. However, we also considered the possibility that, because participants with a CUD would have attenuated DA increases when given MP, they would show a lower than expected reward resulting in inhibition of DA neuronal firing, whereas the expectation of receiving PL and instead receiving MP in CUD would lead to a greater than expected reward signal and hence greater DA neuronal firing than in controls.

## Methods

### Subjects

The Institutional Review Boards at the Stony Brook University/Brookhaven National Laboratory approved the protocol. Written informed consent was obtained after the experimental procedure was explained to the participants. A total of *n* = 23 CUD and *n* = 23 HC participants were recruited from the New York metropolitan area and were matched for age (range: 34–50), gender (4 females in each group), body mass index, education years, and intelligence quotient (IQ) (see Table 1 for demographic details of each group). Participants completed the Full-Scale IQ from the Wechsler Adult Intelligence Scale, and Barona estimates were computed by incorporating race, region of the country in which the person is living, and whether they live in an urban or rural environment^18^. There were also no differences in ethnicity between the CUD (African American: *n* = 18, Caucasian: *n* = 4, mixed ancestry: *n* = 1) and HC group (African American: *n* = 18, Caucasian: *n* = 3, mixed ancestry: *n* = 2; *χ*^2^_46,2_ = 0.5, *p* = 0.8), which has been shown to be associated with D2R^19^. There were, however, more smokers in the CUD (current smokers: *n* = 20, ex-smokers: *n* = 1, never-smokers: *n* = 2) compared to the HC group (current smokers: *n* = 3, ex-smokers: *n* = 1, never-smokers: *n* = 19; *χ*^2^_46,2_ = 26.3, *p* < 0.001). Result on the effects of MP when it was not expected were previously published as part of a larger study that compared the effects of MP between CUD and HC^17^. However, this study is the first in reporting the effect of Expectation on D2R availability in CUD after PL and after MP and in comparing the Expectation responses to PL and to MP between controls and CUD.

**Table 1:** Characteristics of the study participants

	CUD (*n* = 23)	HC (*n* = 23)	*p* Value
Age (years)	44.1 (3.6)	42.1 (4.1)	ns
Years of education	13.2 (5.7)	14.7 (2.8)	ns
Verbal IQ Barona	97.0 (11.9)	97.8 (13.6)	ns
BMI	26.4 (5.0)	26.4 (2.3)	ns
Cigarette (pack years)	8.8 (5.6)	0.5 (1.9)	<0.0001
Cocaine age	21.9 (5.8)	NA	NA
Cocaine use (g/day)	3.5 (2.8)	0	<0.0001
Cocaine use (years)	18.4 (7.2)	0	<0.0001
BDI	10.4 (11.3)	2.1 (2.6)^a^	0.002
MPQ PEM	44.2 (9.3)	51.9 (9.9)	0.010
MPQ NEM	19.5 (11.5)	8.4 (7.0)	<0.0001
MPQ constraint	49.1 (7.9)	55.6 (8.4)	0.010

*BDI* Beck’s depression inventory, *BMI* body mass index, *cigarette (pack years)* (number of cigarettes per day **×** years of smoking)/20, with 20 being the size of a common pack of cigarettes, *CUD* cocaine use disorder, *HC* healthy controls, *MPQ* multidimensional personality questionnaire, *PEM* positive emotionality, *NEM* negative emotionality, *NA* not applicable, *ns* not significant

^a^*n* = 21

CUD subjects were included if they met Diagnostic and Statistical Manual of Mental Disorders IV diagnosis for current cocaine dependence with at least a 2-year history of cocaine abuse (at least 3 g/week), use cocaine predominantly by smoked or intravenous (iv) route, and presently intended to continue using cocaine. The inclusion criteria for the HC subjects were a prior experience of using stimulant (i.e., cocaine, methamphetamine, MP) at least once but no presence or history of other substance use disorders than nicotine. CUD and HC were excluded if they had current or a history of neurological disease of central origin or psychiatric disease including seizures, high levels of anxiety, panic attacks, psychosis; current medical illness that may affect brain function; current or history of cardiovascular disease; head trauma with loss of consciousness >30 min; urine positive for psychoactive drugs; and history of vascular headaches.

### Study design

Participants were scanned with 4 PET scans and [^11^C]raclopride after MP (0.5 mg/kg iv) or PL. Brain DA D2R availability was measured in 2 days under 4 conditions on two different days: (1) PL/PL: expecting PL and receiving PL, (2) PL/MP: expecting PL and receiving MP, (3) MP/PL: expecting MP and receiving PL, (4) MP/MP: expecting MP and receiving MP. The order of sessions was pseudo-randomized over 2 days and equally distributed over diagnostic groups. For practical reasons, receiving MP was always provided after receiving PL (always the second scan on a given day) to avoid the pharmacological effects of MP that would otherwise affect the following scan, providing the following combinations: day A: PL/PL following PL/MP and day B: MP/PL following MP/MP; or day A: PL/PL following MP/MP and day B: MP/PL following PL/MP. MP (0.5 mg/kg) or PL (saline 3.0 ml) was administrated iv for 30 s.

### Drug expectation

Thirty minutes before the injections, participants were told by the experimenter which injection they would receive (PL or MP). Participants were also told that there was a chance that they would be deceived. Participants were not debriefed after their scans to minimize learning effects on the effects of MP or PL.

### Plasma MP concentrations, cardiovascular, and self-report ratings

Blood samples were obtained to measure plasma MP concentration prior to and at 15, 30, 45, and 60 min after MP. The plasma concentration of MP was analyzed at Dr. Thomas Cooper’s laboratory (Nathan Kline Institute, New York). In addition, cardiovascular responses (i.e., heart rate, blood pressure, and EKG lead II) and self-report ratings (i.e., high, rush, anxious) to MP and PL were recorded prior to their injection and then every minute for the first 20 min after injection, then every 5 min at the end of the MP and PL PET scans.

Effects of MP on cardiovascular responses and self-report ratings were tested by comparing the average scores obtained between 0 and 60 min after iv PL and MP administration using a mixed analysis of variance (ANOVA) with Group × Challenge × Expectation in SPSS 22 (IBM, Armonk, NY). Post hoc *t* tests were performed to determine the direction of findings. Cardiac data and self-reports were further correlated with MP-induced changes in striatal non-displaceable binding potential (BP_ND_).

### PET scanning and processing

PET scans were started at 2 min after PL or MP administration for 1 h. Dynamic emission scans were done using the Siemens HR+ tomograph (4.5 × 4.5 × 4.2 mm^3^; three-dimensional acquisition mode) and were started after iv injection of ~7 mCi of [^11^C]raclopride. Twenty dynamic emission scans were obtained from the time of injection up to 60 min, which has been our standard procedure for [^11^C]raclopride scans, and included 15 1-min, 3 5-min and 2 15-min scans. There were 120 min between injections, thus 60 min of break between scans.

We normalized the distribution volume (DV) in each voxel to that in the cerebellum (left and right regions of interest (ROIs)) to compute the DV ratio (DVR) images^20^. DVR images were spatially normalized (2-mm isotropic voxels) to the stereotactic space of the Montreal Neurological Institute (MNI) using a 12-parameter affine transformation and a DVR template and spatially smoothed (full width half maximum, FWHM = 8 mm) in SPM8 (Wellcome Trust Centre for Neuroimaging, London, UK). The DVR template was developed from DVR images of 34 healthy subjects that were acquired with [^11^C]raclopride using the same PET scanning sequence. Specifically, all 34 images were carefully inspected to ensure whole-brain coverage and images with potential artifacts were excluded. A 12-parameter affine transformation with 16 nonlinear iterations was used to register the 34 images to the PD.mnc MNI template provided with the SPM8 package. The images were resliced using the bounding box: *x* = 90:90 mm, *y* = 126:90 mm, and *z* = 72:108 mm and a voxel size of 2 × 2 × 2 mm^3^ using bilinear interpolation, averaged and smoothed (FWHM = 8 mm) to compute BP_ND_ in MNI space, which corresponds to D2R availability^21^. This left each participant with 4 BP_ND_ scans: (1) expecting PL/receiving PL, (2) expecting PL/receiving MP, (3) expecting MP/receiving PL, (4) expecting MP/receiving MP.

### Data analysis

For each participant, difference scans of (receive PL–receive MP) were computed, as a measure of MP-induced DA release, both for the expecting MP and expecting PL conditions. Voxel-wise analyses were performed in SPM8 using a mixed design ANOVA model on these DA release scans, with Group (CUD versus HC) as a between-subject factor, and Expectation (expecting PL versus expecting MP) as a within-subject factor. Corrections for multiple comparisons were performed via the random field theory, an approach that is less conservative than the traditional Bonferroni method. Imaging voxels that were significantly different between conditions were identified using an uncorrected voxel threshold *p* *≤* 0.005 with a minimum cluster size of 100 voxels, and *p* < 0.05 family-wise error (FWE) cluster-corrected. Moreover, for DA regions and their projections targets (caudate, putamen, NAc, midbrain), we created bilateral anatomical masks using Wake Forest University Pickatlas^22^. For each contrast, these masks were used for small-volume correction (SVC) in SPM8 with a significance threshold of *p* < 0.05 FWE, *k* > 10, as described previously^23^. Bilateral mask sizes were the following: caudate (3571), putamen (3592), NAc (313), midbrain (2289).

## Results

### Plasma MP concentrations

Plasma MP concentrations of two HC subjects were missing due to technical failure, leaving 23 CUD and 21 HC for further analyses. For mean MP concentrations, there were no effects of Group (*F*_1,42_ = 0.3, *p* = 0.6), Expectation (*F*_1,43_ = 0.8, *p* = 0.4), and no interaction effect of Group × Expectation (*F*_1,43_ = 0.5, *p* = 0.5) (Table 2). Plasma MP concentrations decreased significantly over time (*F*_3,36_ = 69.7, *p* < 0.0001). MP concentrations decreased faster in CUD than in HC (*F*_3,36_ = 6.3, *p* = 0.002) (see Table 2 for MP plasma values at 15, 30, 45, and 60 min after MP injection in CUD and HC; both for expected and unexpected MP). There were no effects of expectations on MP decrease rates (*F*_3,36_ = 0.6, *p* = 0.6).

**Table 2:** Plasma methylphenidate (MP) concentration (ng/ml) after intravenous MP injection in the CUD and HC in the unexpected (PL/MP) and expected (MP/MP) conditions

	CUD (*n* = 23)	HC (*n* = 21)	*p* Value
PL/MP			
15 min	181.0 (56.7)	161.0 (60.1)	ns
30 min	117.6 (24.1)	136.4 (49.2)	ns
45 min	92.3 (19.9)	106.6 (21.9)	0.03
60 min	79.1 (18.7)	93.4 (20.0)	0.02
Mean	117.6 (25.8)	123.3 (30.0)	0.4
MP/MP			
15 min	182.0 (61.2)	150.1 (53.9)	ns
30 min	114.8 (34.0)	133.2 (41.0)	ns
45 min	91.9 (22.7)	105.6 (19.7)	0.04
60 min	77.8 (16.7)	89.1 (17.4)	0.04
Mean	116.9 (30.2)	119.5 (27.0)	ns

*CUD* cocaine use disorder, *HC* healthy controls, *PL* placebo, *ns* not significant

### Cardiovascular responses to MP

**Fig. 1:**
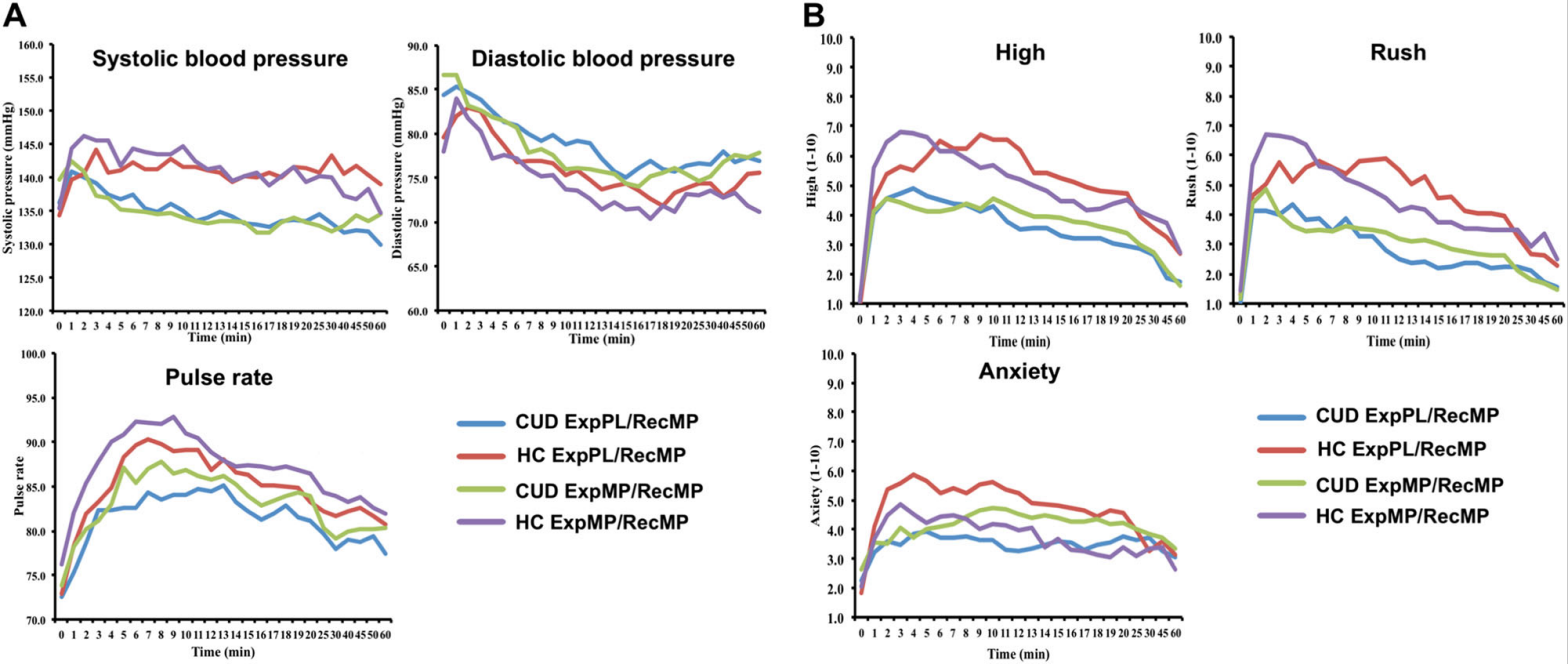
**a** Healthy controls (HC) showed stronger MP-induced increases in pulse rate and systolic blood pressure than cocaine use disorder (CUD). **b** CUD reported lower rush and high compared to HC during the MP challenge. There was no effect on expectation on anxiety when challenged with PL in either group. When challenged with MP, HC had higher anxiety whereas CUD showed reduced anxiety in unexpected condition

MP administration significantly increased cardiovascular responses (pulse rate, systolic and diastolic blood pressure) in both unexpected MP and expected MP conditions, compared to PL (all *p* < 0.0001). There was a main effect of Expectation on pulse rate (*F*_1,44_ = 8.2, *p* = 0.006); with the expectation of MP increasing pulse rate for both the PL challenge (*t*_45_ = 2.0 *p* = 0.05) and the MP challenge (*t*_45_ = 1.9 *p* = 0.07). There were, however, no effects of Expectation × Group, Expectation × Challenge, or Expectation × Challenge × Group. There was a Challenge × Group effect for pulse rate (*F*_1,44_ = 4.9, *p* = 0.032) and systolic (*F*_1,44_ = 10.1, *p* = 0.003) but not diastolic blood pressure (*F*_1,44_ = 1.3, *p* = 0.3), with stronger MP-induced increases in pulse rate (*t*_44_ = 2.2, *p* = 0.03) and systolic blood pressure (*t*_44_ = 3.2, *p* = 0.003) in HC than in CUD. Figure 1a depicts cardiovascular responses to MP in both groups. These findings on cardiovascular responses are highly consistent with an earlier study with iv injection of MP in abstinent cocaine users^24^.

### Behavioral responses to MP

MP challenge increased ratings of “rush,” “high,” and “anxiety” compared to PL (all *p* < 0.0001). There were, however, no main effects of expectation on self-report ratings.

For “rush” and “high,” there was a main effect of Group (rush: *F*_1,44_ = 7.7, *p* = 0.008; high: *F*_1,44_ = 5.9, *p* = 0.02), and an interaction effect of Challenge × Group (rush: *F*_1,44_ = 8.4, *p* = 0.006; high: *F*_1,44_ = 8.1, *p* = 0.007), showing that during the MP challenge CUD reported lower “rush” (*t*_44_ = 2.9, *p* = 0.006) and “high” (*t*_44_ = 2.6, *p* = 0.01) compared to HC, but there were no group differences for PL challenges (*p* > 0.05). For “anxiety” there was a Challenge × Group effect (*F*_1,44_ = 5.9, *p* = 0.02), with higher anxiety ratings during the PL challenges in CUD (mean = 2.3 ± 1.9) compared to HC (mean = 1.4 ± 0.8; *t*_44_ = 2.0, *p* = 0.05), but no differences in MP.

For “anxiety,” there was an Expectation × Group (*F*_1,44_ = 4.5, *p* = 0.04) and an Expectation × Challenge × Group effect (*F*_1,44_ = 7.2, *p* = 0.01). Paired *t* tests showed that there was no effect on Expectation on anxiety when challenged with PL in either group (*p* > 0.18). However, when challenged with MP, controls had higher anxiety when expecting PL (mean = 4.7 ± 2.6) than expecting MP (mean = 3.7 ± 2.1; *t*_22_ = 2.4, *p* = 0.02), whereas CUD showed reduced anxiety when expecting PL than MP at trend level (*t*_22_ = 1.9, *p* = 0.073). Figure 1b depicts self-reported responses to MP in both groups.

## PET results

### Baseline D2R availability between CUD and HC

For iv-PL conditions (baseline), there was a main effect of Group in the bilateral putamen, with CUD showing lower D2R availability than HC (*p* < 0.05 SVC), which is consistent with previous findings (Supplementary Figure 1; Supplementary Table 1). However, there was no main effect of Expectation and no Group × Expectation interaction effect at *p* > 0.05 SVC.

### Effects of MP on DA release as measured by the difference in [^11^C]raclopride BP between the PL and MP conditions

**Fig. 2:**
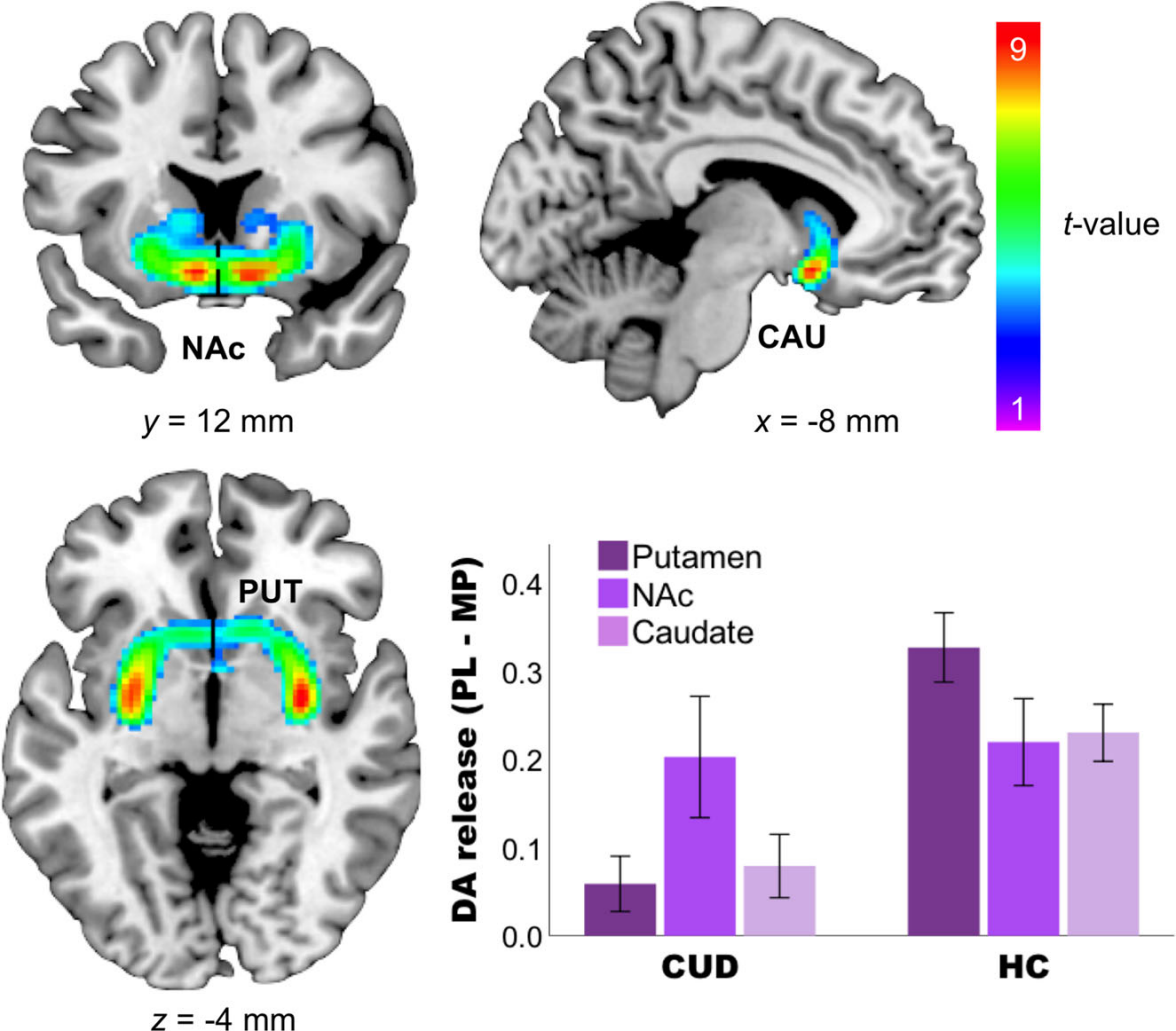
Methylphenidate (MP) increased dopamine (DA) release in striatal areas as measured by the difference between BP_PL_ and BP_MP_. The SPM difference images were superimposed onto T2-weighted magnetic resonance images in coronal (left upper), sagittal (right upper), and transverse (left lower) views. The color bar indicates *t* score values. Red represents the highest value and dark violet represents the lowest value. The bar chart showed DA release (PL–MP) in striatal regions in cocaine use disorder and healthy controls

MP increased DA release in the bilateral putamen (*p* < 0.0001 FWE cluster-corrected), as well as in bilateral ROIs NAc and caudate (all *p* < 0.05 SVC) (Fig. 2; Table 3).

**Table 3:** Whole-brain results of Group × Expectation for *iv-PL* *>* *iv-MP*

Brain region	L/R	*K*	MNI (*x* *y* *z*)			*t* Value
Main effect of Challenge: *iv-PL* *>* *iv-MP*						
Putamen	R	4924	30	−6	−4	11.38^a^
	L		−28	−4	−6	10.88^a^
Caudate	L	756	−8	12	−12	10.30^b^
	R		10	12	−12	8.96^b^
NAc	R	132	10	12	−12	8.96^b^
Group × Challenge: *CUD* *<* *HC, iv-PL* *>* *iv-MP*						
Putamen	R	1086	30	−8	−6	9.74^a^
	L	1110	−28	−8	−6	8.47^a^
Caudate	L	163	−14	14	−12	4.85^b^
	R	209	12	16	6	4.43^b^
NAc	L	48	−14	14	−12	4.85^b^
	R	12	20	12	−12	3.73^b^
Expectation × Challenge: *Expect PL* *>* *Expect MP, iv-PL* *>* *iv-MP*						
No significant voxels						
Group × Expectation × Challenge: *CUD* *>* *HC*, *Expect PL* *>* *Expect MP, iv-PL* *>* *iv-MP*						
Caudate	L	95	−16	−12	22	4.06^b^
Midbrain	L	53	−8	−12	−16	3.64^b^
	R	43	2	−26	0	3.63^b^

*BA* Brodmann area, *CUD* cocaine use disorder, *HC* healthy controls, *iv* intravenous, *K* cluster size, *L* left, *MNI (x y z)* coordinates in Montreal Neurological Institute space (*x* *y* *z*), *MP* methylphenidate, NAc nucleus accumbens, *PL* placebo, *R* right

^a^*p* < 0.05 family-wise error corrected (FWE) whole brain, cluster-corrected

^b^*p* < 0.05 FWE small-volume corrected

### Interaction effects of Group × Challenge × Expectation

**Fig. 3:**
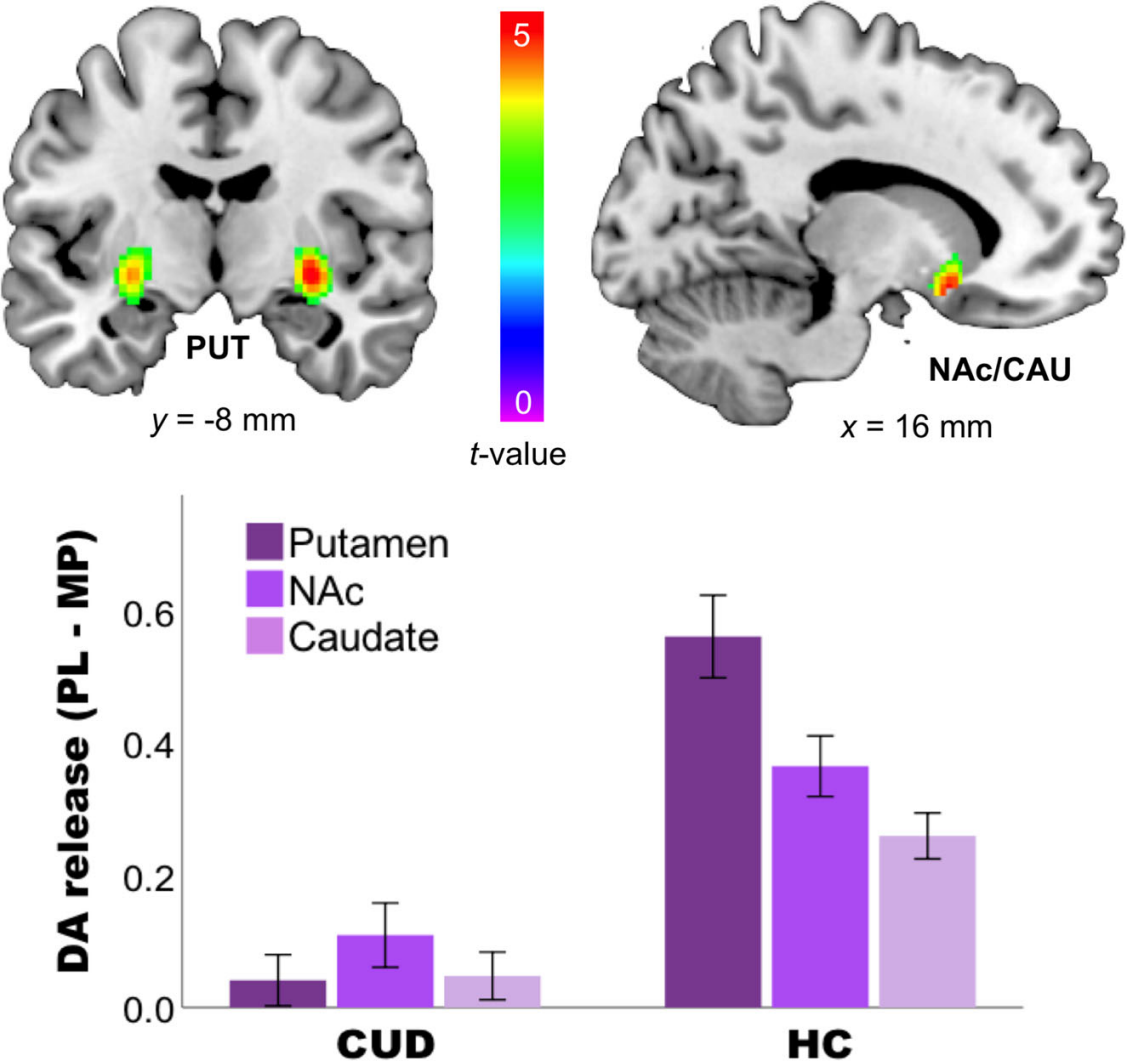
Interaction effects of Group × methylphenidate (MP) Challenge between cocaine use disorder (CUD) and healthy controls (HC). CUD has blunted responses to MP in putamen, caudate, and nucleus accumbens as compared to HC. The SPM images were superimposed onto T2-weighted magnetic resonance images in coronal (left upper) and sagittal (right upper) views. The color bar indicates *t* score values. Red represents the highest value and dark violet represents the lowest value. The bar chart showed DA release (PL–MP) in striatal regions in CUD and HC

**Fig. 4:**
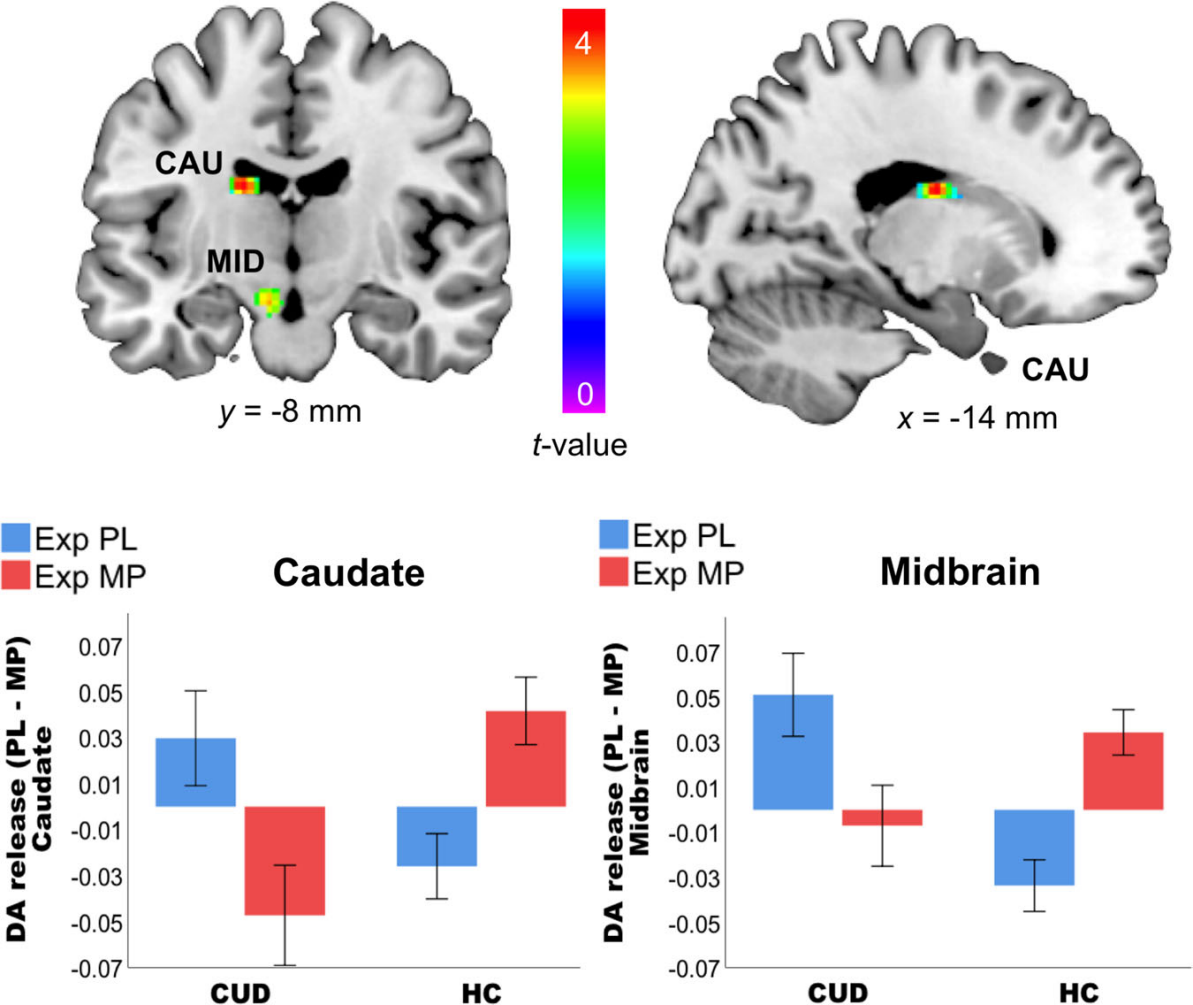
Interaction effect of Group × Challenge × Expectation in the caudate and midbrain (*p* < 0.05 small-volume corrected). The expectation of methylphenidate (MP) increased MP-induced dopamine (DA) release (PL–MP) in healthy controls (HC) but not in cocaine use disorder (CUD) in whom it tended to reduce it, whereas expectation of PL tended to increase DA release in CUD and decrease in HC. The SPM images were superimposed onto T2-weighted magnetic resonance images in coronal (left upper) and sagittal (right upper) views. The color bar indicates *t* score values. Red represents the highest value and dark violet represents the lowest value. The bar chart showed DA release (PL–MP) in caudate and midbrain of CUD and HC

There was a Group × Challenge interaction effect, with putamen, caudate, and NAc (all *p* < 0.05 SVC) showing blunted responses to MP in CUD compared to HC (Fig. 3; Table 3), although the effects of Group × Expectation or Challenge × Expectation were not significant (Table 3). In contrast, there was a Group × Challenge × Expectation effect in caudate and midbrain (*p* < 0.05 SVC; Fig. 4), showing the effects of expectation on MP-induced DA increases to have opposite effects in CUD than in HC in these two regions. Specifically, in HC expectation increased the effects of MP, whereas in CUD it decreased them; in juxtaposition, when expecting PL in HC MP tended to inhibit DA release, whereas in CUD it increased DA.

### Correlations with cardiovascular and self-report ratings

**Fig. 5:**
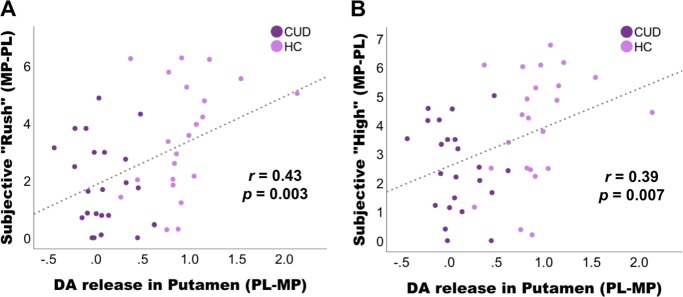
Correlations between dopamine (DA) release in putamen with methylphenidate-induced increases in self-report ratings (from 1 to 10) of “rush” and “high”. **a**, **b** Significant correlations were present for groups pooled together and appear to be predominantly driven by the strong group difference in DA release in putamen (*p* < 0.0001) and ratings of “rush” (*p* = 0.006) and “high” (*p* = 0.007)

Activations for main effect of Challenge (putamen, caudate, NAc) were extracted using SPM8 and were correlated with its cardiovascular and self-reported responses. DA release in putamen correlated positively with MP-induced increases in self-report ratings of “rush” (*r* = 0.43, *p* = 0.003) and “high” (*r* = 0.39, *p* = 0.007) when groups were pooled together (Fig. 5). However, these correlations were not present for each group separately (the correlations reached trend level only in HC: rush: *r* = 0.39, *p* = 0.06, high: *r* = 0.32, *p* = 0.14)) and thus are largely driven by the strong group difference in DA release in putamen (*t* = 8.8, *p* < 0.0001) and ratings of “rush” (*t* = 2.9, *p* = 0.006) and “high” (*t* = 2.8, *p* = 0.007) during MP–PL conditions. Group × Challenge × Expectation interaction values in caudate or midbrain did not correlate with interactions in behavior or cardiovascular responses.

## Discussion

Our study replicated previous findings in showing lower D2R availability^25,26^ and blunted striatal DA release in CUD compared to HC and is consistent with prior reports of attenuated DA responses to MP in active^17^ and detoxified CUD^25^ and with attenuated DA increases to amphetamine in detoxified CUD^26^. The study further showed a Group × Challenge × Expectation effect in the left caudate and bilateral midbrain (*p* < 0.05 SVC). More specifically, it showed opposite effects in HC and CUD such that expectation of receiving MP increased and decreased MP-induced DA release in HC and CUD, respectively; in juxtaposition, the expectation of receiving PL enhanced DA release in CUD but reduced it in HC. This finding was contrary to our hypothesis that CUD would show conditioned responses to expectation of MP hence an amplified effect, similar to our previous study in which we showed overall elevated brain glucose metabolism responses to expectation of MP in CUD versus HC^4^. This discrepancy is likely to reflect the fact that brain glucose metabolic responses reflect not just DA effects but also downstream DA responses as well as non-DA responses implicated in conditions (i.e., glutamatergic modulation)^27^.

The DA system is critical for signaling expectation of rewards^28^ and for motivating behaviors needed to achieve the reward^15^. DA neurons in midbrain initially respond to unexpected primary rewards and with repeated exposure eventually to the stimuli that predict those rewards^29^. The anticipation of a rewarding outcome is often inferred based on activation in striatal regions, including caudate, putamen, NAc^30–32^, and in ventral cingulate and orbitofrontal cortices^8,33^. Neurons in the midbrain code for a breach in expectation (i.e., prediction error)^32^. The activation is associated with a distributed network involved in detecting, signaling, and adjusting behavior and expectations toward violated prediction^34^. Caudate neurons have been shown to participate in feature-based anticipation of visual information that predicts reward. Caudate neurons fire not only before the onset of an expected target but also in preparatory responding to signals that predict reward^35^. When predictions are violated, caudate activity attenuates over the course of learning. Once the rules have been established, breaches of expectation diminish and so does the caudate activity. The caudate signals for the occurrence of events that violate the predictions^36^. This signal is not due to the perception of salient events or the need to change one’s behavior. Thus one interpretation of our findings is that in CUD the blunted DA and self-reported responses to MP was coded as an omission of reward that led to decrease DA cell firing and reduced DA release in caudate and midbrain consistent with the role of DA encoding for reward prediction error. That is, DA neurons are activated when the reward is larger than predicted, whereas they are inhibited when the reward is less than predicted^28,37^. This could also explain why an unexpected signal with PL might have led to increased DA release in CUD who might have responded to conditioning to the act of injection, which would have not been the case for HC. Moreover, recent studies in healthy adults showed that MP decreased midbrain connectivity with the putamen^38,39^.

Previous PET studies in healthy volunteers have shown that the expectation of stimulant drugs (e.g., MP, amphetamine, and caffeine), without active drug administration, is associated with dopaminergic PL effects, including D2R reductions in the striatum and thalamus^8,40,41^. Moreover, PL analgesia has also shown increased striatal and extra-striatal dopaminergic responses under the expectation of analgesia in various studies with human pain models^5,42,43^, and patients with Parkinson’s disease who clinically responded to PL treatment showed greater striatal DA release compared to non-responders^6,7^. A recent [^11^C]raclopride study compared dopaminergic responses to alcohol and PL beverages in AUD patients and in healthy participants with and without a family history of AUD. They found a drink order-by-group interaction such that the family history positive group who received PL first had lower PL BP and lower difference between PL and alcohol BP, which was in line with their subjective expectation of alcohol evoking DA release^13^. Moreover, in tobacco smokers, the belief of “absence of nicotine in a cigarette” compared to “presence of nicotine in a cigarette” diminished neural blood oxygen-level-dependent responses in the ventral striatum to value and reward prediction errors and reduced its impact on smokers’ choices^11^. However, the evidence for the conditioned DA responses to the expectation of drugs of abuse in drug abusers is scarce, and we based our hypothesis mainly on a previous brain glucose metabolism study in CUD^4^ and to brain cue reactivity experiments in which CUD show conditioned limbic responses to cocaine cues^44,45^. Potentially, our unexpected findings of blunted DA effects of MP expectation in CUD are due to the administration of MP. MP is a psychostimulant drug that, similarly to cocaine, increases DA by blocking DATs, and it is distinct from cocaine in its route of administration (iv injection (MP) versus smoking (cocaine)) and drug effects. Thus expected conditioned responses to MP in CUDs may only occur if CUD expect to receive cocaine and not MP.

We further found that DA release in putamen correlated with subjective “rush” and “high” across all subjects. This is in line with previous studies that drug-induced increases in DA are related to subjective “high”: that is, increases in DA release plus faster drug uptake elicit more intensive highs^46^. Large and fast increases of DA mimic natural phasic increases in DA release, which are related to reward and salience^28^. However, these correlations were not present for each group separately (only at trend level in controls) and thus were predominantly driven by the strong group differences in DA release in putamen and in the self-reports of “rush” and “high.” Moreover, studies on expectation effects have also shown that arousal ratings during PL (expected caffeine) are associated with [^11^C]raclopride binding in putamen^41^, and expected “high” was associated with a greater MP-induced metabolic increase in the thalamus in cocaine abusers^4^. In our study, however, expected DA release did not correlate with self-report ratings. Also because MP increases both dopaminergic and noradrenergic signaling, it is possible that some of the changes in self-report for drug effects such as increases in anxiety might have been mediated by noradrenergic effects rather than dopaminergic mechanisms.

The findings from our study are clinically relevant since the difference between the expected outcome and the actual pharmacological effects of the drug in addicted individuals, which are markedly attenuated, could contribute to compulsive drug consumption in an attempt to achieve the expected outcome^15^. Our findings documenting that individuals with a CUD showed reduced DA responses to MP when expecting it but enhanced responses when expecting PL (opposite to the responses in controls) support the model that dopaminergic responses reflect the predicted expectation of reward^47^ and document for the first time that these DA responses are altered in addiction when compared to controls. To the extent that therapeutic interventions can be developed to restore to normal the reward prediction error signals in addicted individuals, it might be possible to prevent the craving and the compulsive drive for drug intake in CUD.

Although PET [^11^C]raclopride measures of MP-induced DA changes in the striatum (including caudate) show good test–retest reliability^48^, the reproducibility in midbrain has not been tested, which is a limitation to our study. Also, while in caudate and putamen D2R availability predominantly reflects D2 receptors, in NAc and midbrain they reflect both D2 and D3 receptors. In CUD, there is evidence that D2R are downregulated, whereas D3 receptors might be upregulated^49^, and this therefore confounds the interpretation of our findings. Another limitation of the study is that CUD consisted of more smokers (*n* = 20 current smokers) than the HC group (*n* = 3 current smokers), and group findings may be in part due to group differences in smoking^50^. However, an exploratory analysis of main effects of Challenge in striatal areas (NAc, caudate, putamen), for which we hypothesized blunted DA release in smokers, did not reveal a significant effect of smoking status (*p* > 0.05; corrected for CUD Group). Effects of tobacco smoking on stimulant-induced DA release may not be as strong compared to that of CUD^50,51^. Since CUD is more prevalent in males than in females^52^, recruitment for females was harder than for males, and we matched four women CUD with four HC in our dataset. Haltia et al.^53^ found that iv PL with the expectation of receiving glucose lead to decreased ventral striatal D2R (and increased D2R binding in the dorsal striatum) in men but not in women, suggesting gender differences in DA function after pharmacological challenge^54^. An exploratory analysis revealed no gender effect on striatal DA release in NAc and caudate (*p* > 0.05; corrected for CUD Group). Additionally, although we used a full factorial design of Expectation (PL/MP) × Drug Challenge (PL/MP), which is the golden standard for a PL design, we have not systematically tested whether the deception was successful by asking participants after their scans how certain they were about their drug expectation. Therefore, group differences in sensitivity to deception may have played a role in the outcome. Nevertheless, if a Group effect in deception was indeed present this would be an effect consistent over all four conditions and corrected by the factorial model. Last, we did not correct our analyses for potential sleep disturbances in CUD, whereas it has been previously found that cocaine abusers show shorter sleep durations than non-abusing volunteers^55,56^ and that sleep deprivation reduces striatal D2R in healthy volunteers^57,58^.

In summary, these results in active CUD subjects expanded prior findings of decreased striatal DA responses in detoxified CUD. They also identify group differences in expectation responses in caudate and midbrain such that HC showed DA increases with expectation of receiving MP, whereas CUD did not, which is consistent with dysfunction of caudate and midbrain DA activity.

## Supplementary information

Supplementary Figure1

Supplementary Table1

Supplementary Material
