# Supplementary material for: Expectation effects on brain dopamine responses to methylphenidate in cocaine use disorder
Source: Transl Psychiatry. 2019 Feb 15;9:93. doi: 10.1038/s41398-019-0421-x (PMC6377670; doi:10.1038/s41398-019-0421-x)
Supplement: Supplementary file 1 — Supplementary Figure1 [file 41398_2019_421_MOESM1_ESM.docx]

**Supplementary Material**

**Supplementary Figure 1**. Baseline D2R availability in CUD and HC

At baseline, CUD showed lower D2R availability in Putamen compared to HC (*p*<0.05 SVC). The SPM images were superimposed onto T2 weighted MR images in coronal (left upper), sagittal (right upper) and transverse (left lower) views. The color bar indicates *t*-score values. Red represents the highest value and dark violet represents the lowest value. The bar chart showed D2R availability in Putamen in CUD and HC.


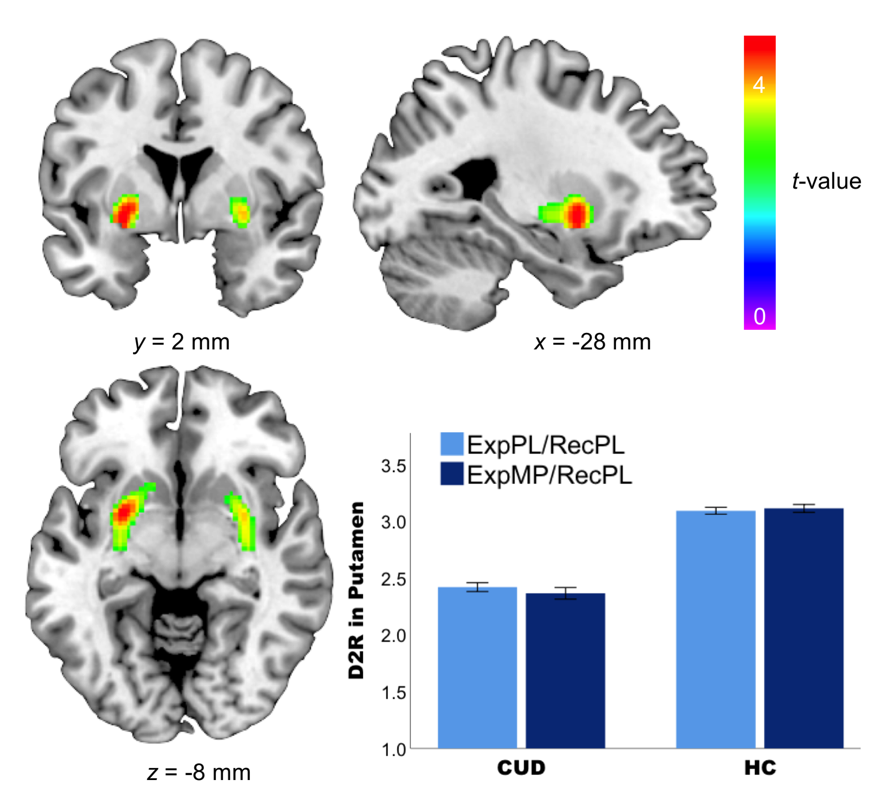


**Supplementary Table 1**. Whole brain results of Group × Expectation at baseline

| **Brain region** | **BA** | **L/R** | **K** | **MNI (x y z)** | | |  | **t-value** |
| --- | --- | --- | --- | --- | --- | --- | --- | --- |
| **Main effect of Group: *HC>CUD*** | | | | | | | | |
| Putamen  Putamen |  | L  R | 397  284 | -28  30 | 2  2 | -8  -4 |  | 4.68^*^  3.88^*^ |
| **Main effect of Expectation: *Expect PL>Expect MP*** | | | | | | | | |
| No significant voxels |  |  |  |  |  |  |  |  |
| **Group × Expectation: *HC>CUD*, *Expect PL>Expect MP*** | | | | | | | | |
| No significant voxels |  |  |  |  |  |  |  |  |

^*^ p<0.05 FWE SVC

Abbreviations: BA = Brodmann Area; CUD = Cocaine Use Disorder; FWE = family-wise error corrected; HC = Healthy controls; Hemi = Hemisphere; iv=Intravenous; K = Cluster Size; L = Left; MNI (x y z) = Coordinates in Montreal Neurological Institute space (x y z); MP = Methylphenidate; PL = Placebo; R = Right; SVC = Small-volume corrected.
