# Supplementary figures and images for: Expectation effects on brain dopamine responses to methylphenidate in cocaine use disorder
Source: Transl Psychiatry. 2019 Feb 15;9:93. doi: 10.1038/s41398-019-0421-x (PMC6377670; doi:10.1038/s41398-019-0421-x)

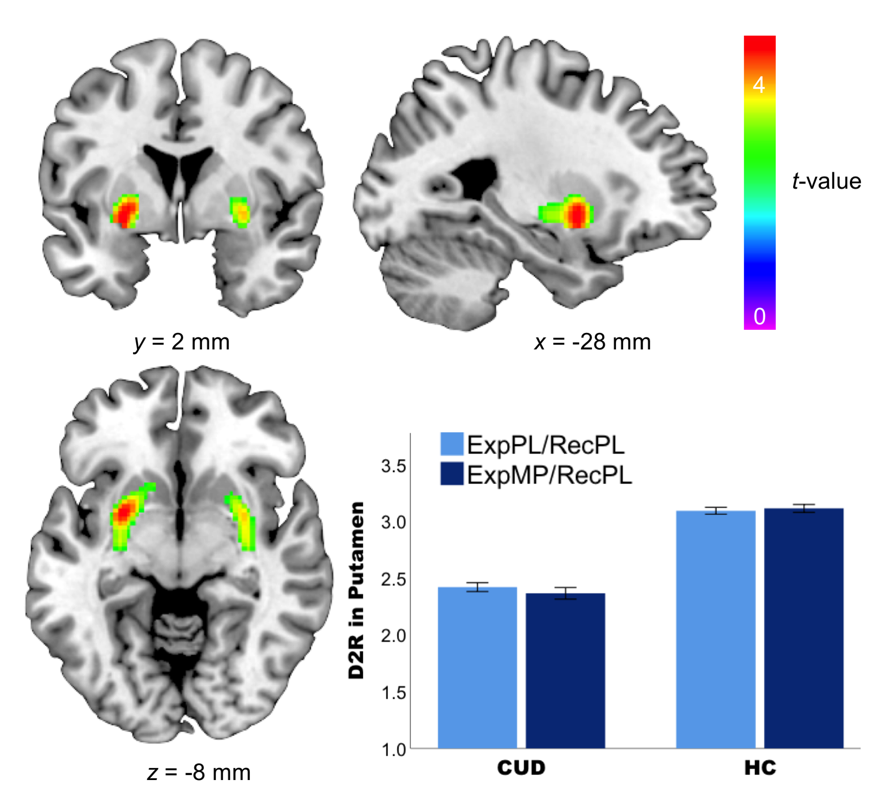

Supplement: Supplementary file 2 — Supplementary Table1 [file 41398_2019_421_MOESM2_ESM.docx]
